# Integrated Analysis of miR-7-5p-Related ceRNA Network Reveals Potential Biomarkers for the Clinical Outcome of Gastric Cancer

**DOI:** 10.1155/2022/8204818

**Published:** 2022-02-14

**Authors:** Meng-Yang Shi, Yan-fei Mu, Neng Shen

**Affiliations:** Chongqing Key Laboratory of Translational Research for Cancer Metastasis and Individualized Treatment, Chongqing University Cancer Hospital, Shapingba District, Chongqing, China

## Abstract

Gastric cancer (GC) is the second leading cause of tumor-associated death and the fourth most commonly seen tumor across the world. Abnormal ncRNAs have been verified to be involved in potential metastasis via modulating epithelial-to-mesenchymal transition progression and are vital for the progression of cancers. Tumor-infiltrating immune cells (TICs) are a vital indicator of whether cancer patients will benefit from immunotherapy. Nonetheless, the association between ceRNAs and immune cells remained largely unclear. We used the ceRNA network combined with TICs for the prediction of the clinical outcome of GC patients based on TCGA datasets. The percentage of immunocytes in GC was speculated by the use of CIBERSORT. Via Lasso and multivariate assays, prognostic models were established applying survival-related genes and immune cells. Nomograms were developed, and the accuracy of the nomograms was determined using calibration curves. The association between ceRNAs and TICs was validated by the use of integration analysis. In this study, there were 2219 mRNAs (1308 increased and 911 decreased), 171 lncRNAs (51 decreased and 120 increased), and 123 miRNAs (55 decreased and 68 increased) differentially expressed between tumor groups and nontumor groups. Five lncRNAs, six miRNAs, and 64 mRNAs were used for ceRNA network construction. Eight genes including LOX, SPARC, MASTL, PI15, BMPR1B, ANKRD13B, PVT1, and miR-7-5p were applied for the development of the prognostic model. Survival assays suggested that tumor cases with high risk exhibited a shorter overall survival. In addition, we included T-cell CD4 memory activated, monocytes, and neutrophils for the development of a prognosis model. Eventually, our team demonstrated the possible associations between the ceRNA prognosis model and prognostic model based on immune cells. To sum up, the ceRNA network could be used for gene regulation and predict clinical outcomes of GC patients.

## 1. Introduction

Gastric cancer (GC) is one of the most commonly seen malignancies, which has approximately one million new cases diagnosed annually [1]. GC has an especially high prevalence in Asia [2]. Even with recent improvements in surgery and chemotherapy, GC remains with a very high morbidity and mortality because most patients are diagnosed at an advanced stage and accompanied by malignant proliferation and extensive lymphatic metastasis [3, 4]. Hence, it is imperative to further understand the biomechanism beneath GC and identify new diagnosis/prognosis markers in this regard.

Long noncoding RNAs (lncRNAs), without protein-coding functions, have aroused more and more academic interest in recent years [5]. These RNAs are vital for diverse BP, especially various cancers [6]. The ceRNA assumption hypothesized that apart from the traditional functions, miRNAs targeting RNAs, a reversed logic exists [7, 8]. Growing evidence has demonstrated that lncRNAs, miRNAs, and mRNAs exhibited important regulatory functions in the development and progression of various tumors [9, 10]. Importantly, many ncRNAs have been demonstrated to regulate tumor metastasis via the EMT pathway. Based on the theory of ceRNA, lncRNA and mRNA may have the identical miRNA response elements. Thus, when miRNAs bind to miRNA response elements on lncRNAs, the expressions of mRNAs may not be suppressed, which could promote tumor progression [11, 12]. More and more researchers have suggested that the exploration of RNA interactions is very important for the improvements of the treatments of various tumors [13, 14]. Based on the above findings, specific lncRNA-miRNA-mRNA ceRNA networks have been developed for many kinds of cancers [15, 16]. However, at the genome-wide scale, the studies involved in comprehensive assays between miRNAs and lncRNAs were rarely reported. So, this research intended to construct a risk-assessment model for the prediction of the clinical outcome of GC patients.

## 2. Materials and Methods

### 2.1. Data Collection and Processing

The stomach adenocarcinoma (STAD) transcriptomic data of HTseq-count were acquired on July 4, 2021, from TCGA database, involving mRNA, lncRNA, and miRNA expression patterns of 375 STAD tissues and 32 noncancerous tissues. Corresponding clinical information including sex, age, survival time, and survival status was downloaded from TCGA on the same day. The abundance data of tumor immune infiltration were obtained from the CIBERSORT database, containing 22 types of immunocytes. Genetic expression information of STAD sufferers and tumor immune infiltration abundance data were combined for obtaining the immune cell infiltration abundance of STAD patients.

### 2.2. Differentially Expressed RNAs' Screening and ceRNA Network Construction

After pooling unmatched probes and calculating the average value when the same RNA was detected multiply, 35668 RNAs including 19064 mRNAs, 14086 lncRNAs, and 2518 miRNAs were used for further analysis. Then, 35668 RNAs, including 19064 mRNAs, 14086 lncRNAs, and 2518 miRNAs, were used for further analysis. “Deseq2” package was used to screen differentially expressed RNAs [17]. |Log2 FCs| > 1 and modified *p* < 0.05 had significance on statistics. The visualization of these RNA differential expressions was realized by “pheatmap” package and used for constructing the ceRNA network. The ceRNA network, showing the interactions between lncRNA-miRNA and miRNA-mRNA, was established via GDC RNA Kits and visualized by Cytoscape software 3.8.0 [18].

### 2.3. Prognostic Model of Survival-Associated Genes in the ceRNA Network

Univariate assays were applied to screen survival-associated genes within the ceRNA network, aiming to prevent the model from overfitting. Thereafter, the Lasso regressive analysis was completed. A prognostic model, via multivariate Cox regressive analyses, was constructed to forecast the survival rate of STAD patients. According to the RS computed by the prognosis model, sufferers were separated into the risk_high_ group and risk_low_ group to explore the survival difference. The accuracy of the model was tested by ROC and the correction curves in the “survival” R package.

### 2.4. Estimation of Immune Cell Infiltration

The 22 types of immunocyte classes in STAD were evaluated by the CIBERSORT algorithm. The specimens with CIBERSORT results of *p* < 0.05 were utilized for analyses. The relative abundance of immunocytes in STAD sufferers was calculated. The correlations of immune cells were estimated. To compare the diverse ICI in tumor and normal samples, the Wilcoxon rank-sum test was used.

### 2.5. Survival Analyses and Prognostic Model of Prognosis-Associated Tumor Immune Cells

K-M analyses were performed for every kind of immune cells to find survival-associated immune cells. Immunocytes with *p* < 0.05 were used for further analyses. Prognosis-associated infiltration immunocyte classes were subjected to selection via univariable Cox regressive analyses. Lasso regressive analyses were utilized to ensure the most proper immune cell types for model construction. A model was established via the regressive coefficients of multivariable Cox regressive analyses. The risk scores separated sufferers into the risk_high_ group and risk_low_ group to explore the survival difference. The ROC and the correction curves were used to identify the nomogram's accurateness.

## 3. Statistical Analysis

All analyses were conducted by Perl 5.30.1 (Holland, MI, USA) and R 3.6.2 (R Core Team, Boston, USA).

## 4. Results

### 4.1. Identification of Differentially Expressed Genes and Construction of the ceRNA Network

**Figure 1:**
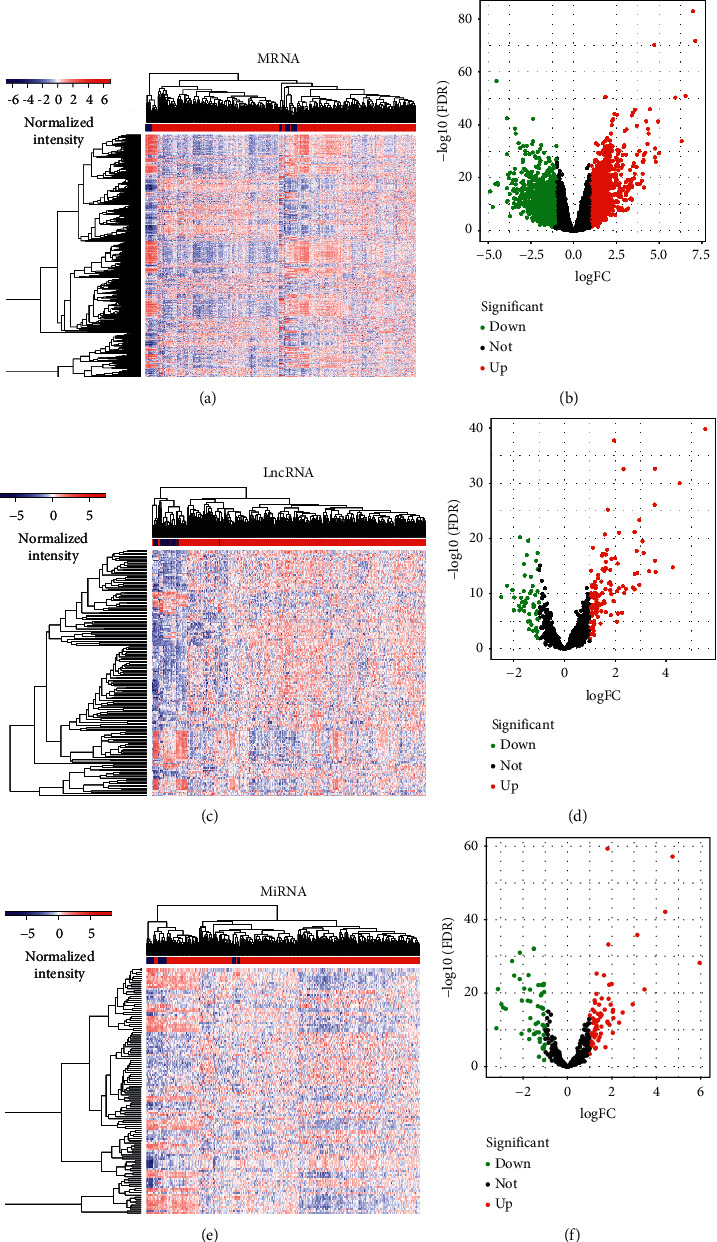
Thermographs and volcano plots of RNA differential expression. (a, b) mRNAs, (c, d) lncRNAs, and (e, f) miRNAs. Red dots represented RNAs with an increased expression, and green dots represented RNAs with a decreased expression.

**Figure 2:**
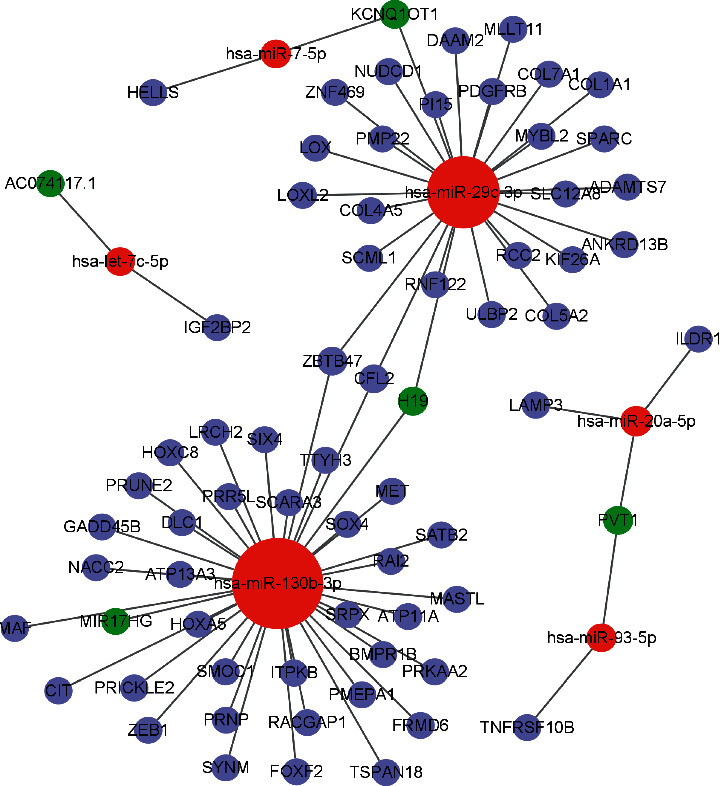
Competing endogenous RNA (ceRNA) network was established by Cytoscape for differentially expressed lncRNA-miRNA-mRNA.

There were 2219 mRNAs (1308 downregulated and 911 upregulated) (Figures 1(a) and 1(b)), 171 lncRNAs (51 downregulated and 120 upregulated) (Figures 1(c) and 1(d)), and 123 miRNAs (55 downregulated and 68 upregulated) differentially expressed between experiment and control groups (Figures 1(e) and 1(f)). Then, the relationships between miRNA-mRNA and lncRNA-miRNA were calculated, and RNAs without regulation relationships were excluded. Finally, 5 lncRNAs, 6 miRNAs, and 64 mRNAs were used for ceRNA network construction. Eventually, we identified 74 edges and 75 nodal points within the network (Figure 2).

### 4.2. Identification of Prognosis-Associated Genes within the ceRNA Network and Establishment of a Prognosis Model

**Figure 3:**
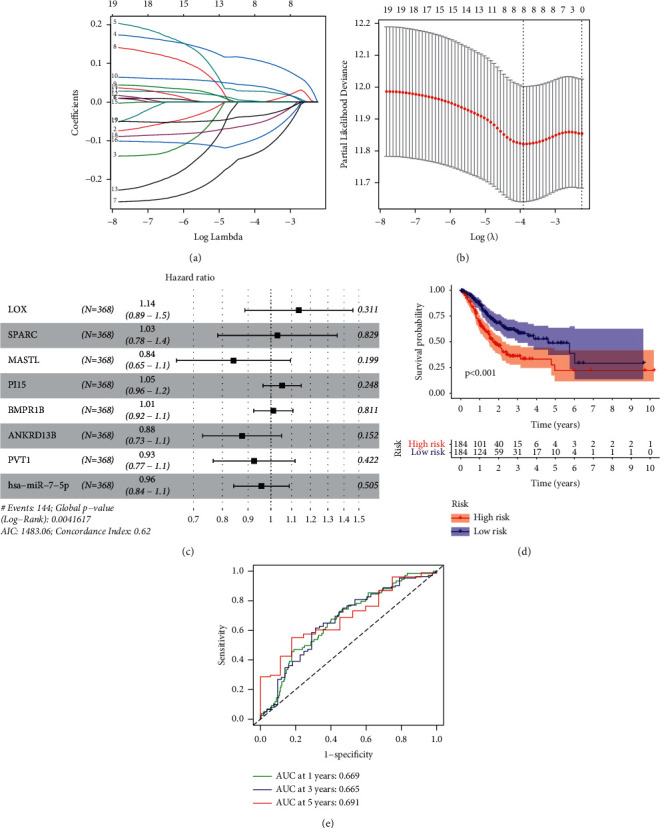
The results of Lasso regression and the model constructed by multivariate assays. (a) Lasso coefficient profiles of the 8 genes related to prognoses from the key members of the ceRNA network. (b) The Lasso regression model for partial likelihood deviance of variables. (c) Multivariate assays of 8 prognosis-associated genes for the development of the prognosis model. (d) Survival assays of patients in the risk_high_ group and risk_low_ group based on the model. (e) ROC curve was employed to verify the model accuracy.

**Figure 4:**
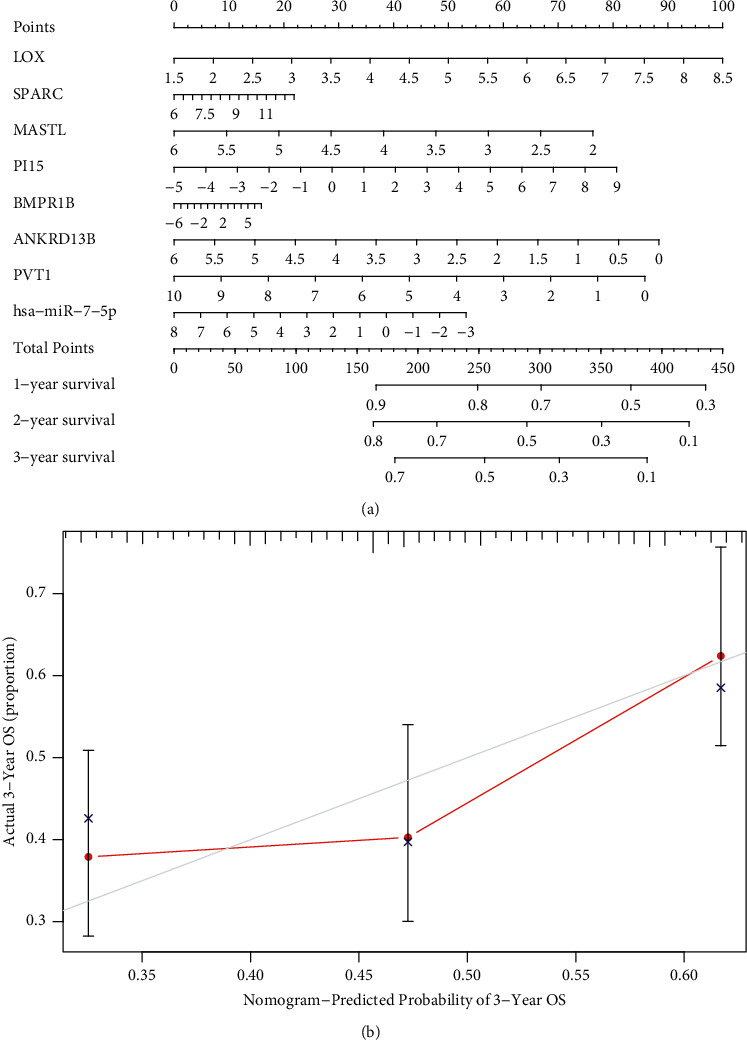
Nomogram constructed as per the model. (a) Nomograph was developed for the prediction of cases' survivals. (b) Calibration curve of the nomogram.

After combining the survival data (status and time) and the gene expression data, we performed univariable Cox regressive analyses and found 19 genes related to survival. To avoid overfitting, we performed Lasso regression analysis (Figures 3(a) and 3(b)). Eight genes (LOX, SPARC, MASTL, PI15, BMPR1B, ANKRD13B, PVT1, and hsa-miR-7-5p) were involved in a Cox proportion risk model used to assess prognosis results (Figure 3(c)). The sufferers were separated into the risk_high_ group and risk_low_ group on the foundation of the model. The survival analyses revealed that the risk_high_ sufferers displayed poorer OS (*p* < 0.001) (Figure 3(d)). The ROC curve revealed that the AUC of 1-year survival registered 0.669, AUC of 3-year survival registered 0.665, and AUC of 5-year survival registered 0.691, suggesting an acceptable accuracy of this model (Figure 3(e)). A nomograph was drawn to forecast the 1-year, 2-year, and 3-year OS potential of STAD sufferers on the foundation of this model (Figure 4(a)). The calibration curve also showed an acceptable accuracy of this nomogram (Figure 4(b)).

### 4.3. Tumor Immune Infiltration

**Figure 5:**
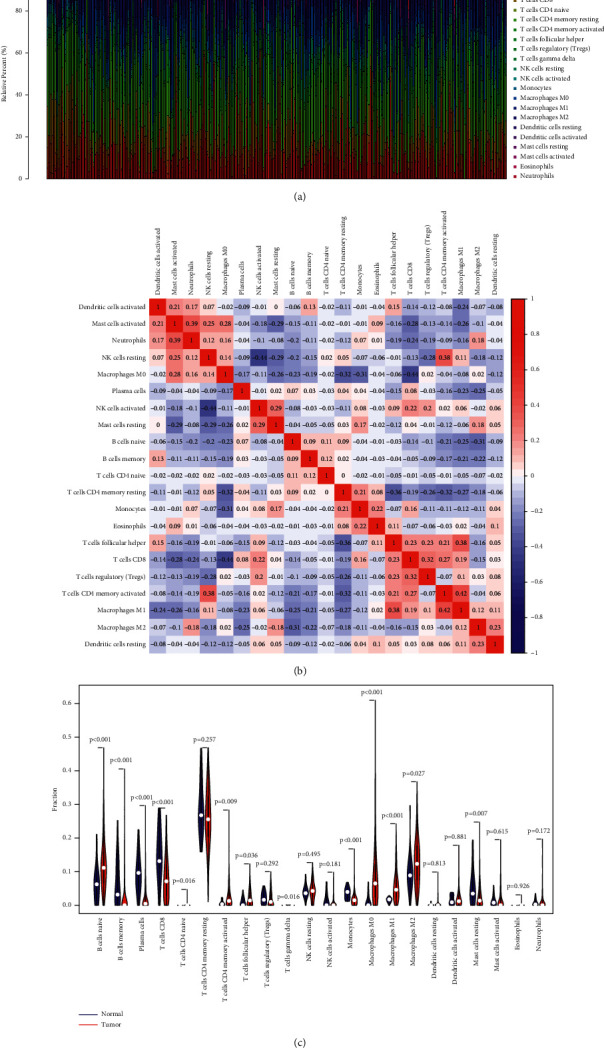
TIICs in the tumor tissues. (a) The relative percentage of 22 TIICs in STAD patients. (b) The correlation of 22 types of TIICs in STAD sufferers. (c) Diversity in the percentage of 22 TIICs between cancer specimens and nontumor specimens.

The relative percentage of cancer-infiltrating immunocytes in STAD patients was evaluated via CIBERSORT arithmetic (Figure 5(a)). The correlations of immune cells were calculated (Figure 5(b)). Compared with normal tissues, B-cell naïve, T-cell CD4 memory stimulated, Tfh, macrophages M0, macrophages M1, and macrophages M2 were highly expressed in STAD tissues significantly. Meanwhile, B-cell memory, plasmic cells, T-cell CD8, monocytes, and mast cells resting were lower expressed in STAD tissues significantly (Figure 5(c)).

### 4.4. Determination of Prognosis-Associated Immune Infiltration Cells and Construction of a Prognosis Model

**Figure 6:**
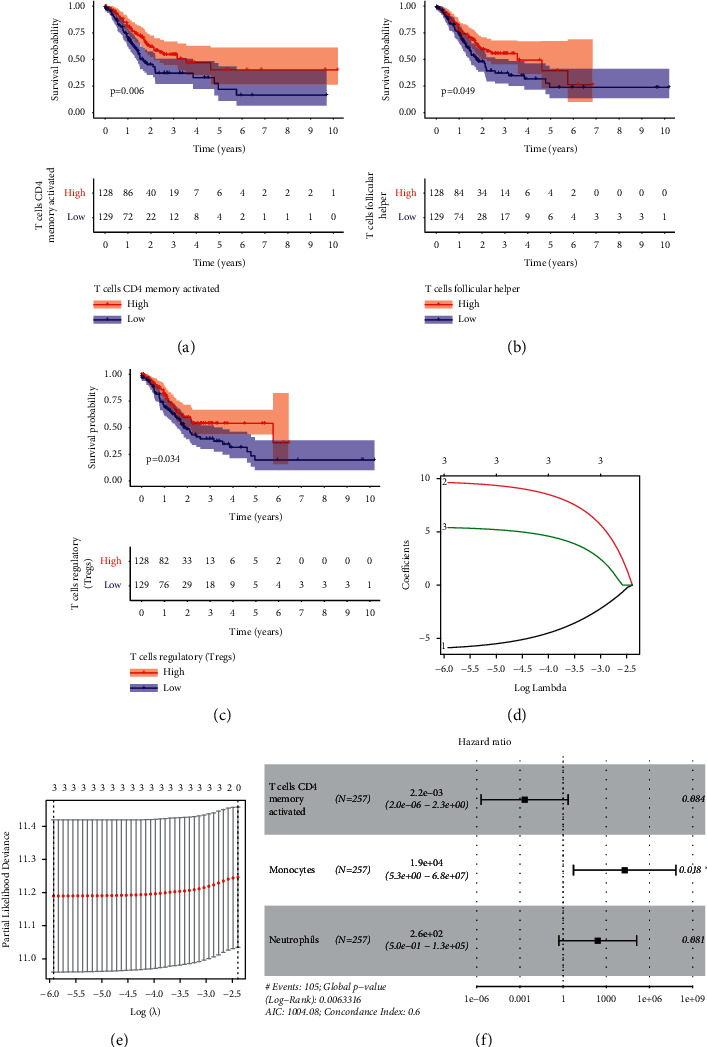
Identification of survival-associated infiltration immunocytes and construction of a prognosis model. (a–c) Survival assays of patients with different proportions of T-cell CD4 memory stimulated, Tfh, and Tregs via Kaplan–Meier analysis. (d) Lasso coefficient profiles of the 22 TIICs. (e) The Lasso regression model for partial likelihood deviance of variables. (f) Multivariate assays of infiltrating immune cells.

**Figure 7:**
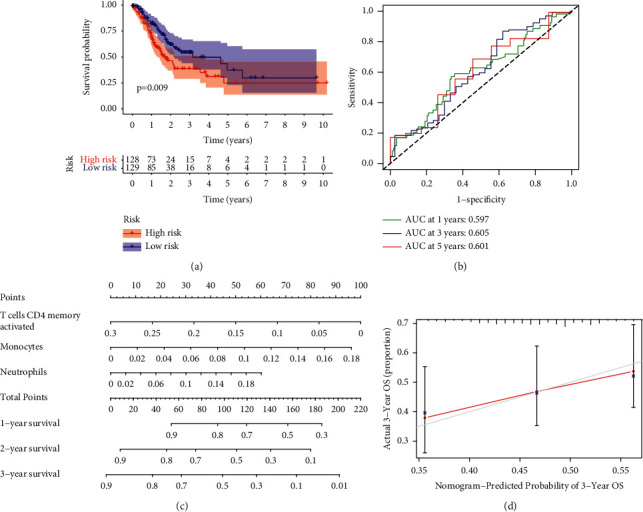
Results of the TIIC prognostic model. (a) Survival assays of patients in the risk_high_ group and risk_low_ group. (b) ROC curve was employed to identify the model accuracy. (c) Nomogram was employed to forecast sufferers' survival time. (d) Correction curve of the nomograph.

Through K-M assays, our group observed that patients with higher proportions of T-cell CD4 memory stimulated, Tfh, and Tregs had better survival (Figures 6(a)–6(c)). Lasso regression and univariable Cox regressive analysis were completed to determine prognosis-associated infiltrating immune cells. The results suggested that T-cell CD4 memory stimulated, monocytes, and neutrophils had better abilities in forecasting prognoses (Figures 6(d)–6(f)). Afterwards, a prognostic model was developed by the use of multivariate assays. On the foundation of the model, sufferers were separated into risk_high_ and risk_low_ groups. Compared with the low-risk group, risk_high_ sufferers displayed shorter survival (*p*=0.009) (Figure 7(a)). The model accurateness was identified by the ROC curves (Figure 7(b)). On the foundation of the model, we constructed a nomograph to forecast the 1-year survival, 2-year survival, and 3-year survival of STAD sufferers (Figure 7(c)). Our team found satisfactory accurateness of such a nomograph was realized through the calibration curve (Figure 7(d)).

### 4.5. Association between Genes and Immunocytes for Prognosis

**Figure 8:**
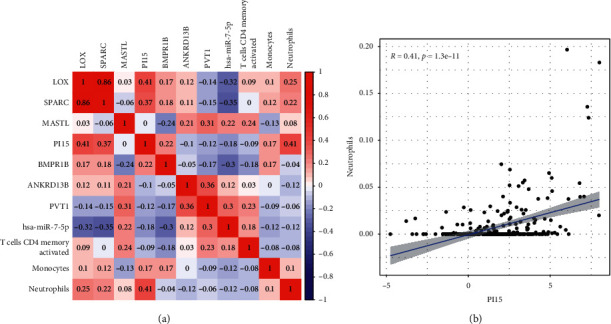
Correlation between the ceRNA prognosis model and infiltration immunocyte prognosis model. (a) The correlation matrix of these models. (b) The association between neutrophils and PI15.

A correlation test was conducted to investigate the relationship between the 8 genes and 3 infiltration immunocytes used for constructing prognostic models (Figure 8(a)). We found that neutrophils were positively associated with PI15, indicating that patients with higher PI15 expression would have more neutrophil infiltration in tumors (Figure 8(b)). For STAD patients, PI15 expression and neutrophil infiltration may be effective prognostic biomarkers.

## 5. Discussion

GC is still a primary public health concern as the 4^th^ most commonly seen tumor and the 2^nd^ leading cause of tumor mortality across the globe [19]. The latest development in genomic, proteomic, and metabolomic techniques has discovered pivotal molecule events in the course of GC carcinogenesis [20]. The findings give rise to the identification of new GC markers, such as gene and epigenesis variations, mRNA, ncRNA, posttranslation protein modification, and metabolin [21, 22]. ceRNA modulatory networks have become hotspots in tumor studies. Previously, several studies have reported the potential of a prognostic model based on the ceRNA network in several types of tumors [23, 24]. In this study, we analyzed TGCA datasets and screened dysregulated lncRNA, miRNA, and mRNA. Then, we developed the ceRNA network of differentially expressed RNAs. Based on the above results, we acquired a prognosis model on the foundation of eight genes (LOX, SPARC, MASTL, PI15, BMPR1B, ANKRD13B, PVT1, and miR-7-5p). Survival assays confirmed that risk_high_ sufferers exhibited undesirable prognoses. ROC assays further confirmed the prognosis significance of our model in GC sufferers, highlighting the potential of our model used as a novel prognostic system.

Previously, the function of the eight genes has been reported in GC. For instance, the expression of miR-7-5p was reported to be low in GC stem cells, and its overexpression distinctly suppressed the growth and invasion of GC stem cells via increasing Smo and Hes1 [25]. SPARC belongs to the matricellular family of secreted proteins. Previous several studies reported that SPARC expression was distinctly increased in gastric cancer, and its knockdown suppressed the metastasis and EMT progress of GC cells [26]. In addition, PVT1, an overexpressed lncRNA in GC, was demonstrated to promote the proliferation and the development of multidrug resistance [27]. Their findings suggested the above genes acted as tumor promotors or suppressors in GC progression, which explained the reason that patients with high risk showed a shorter overall survival. Then, to further forecast the prognoses of GC sufferers at diverse years posterior to diagnoses, our team established a novel nomograph on the foundation of genetic expression. The greater the overall scoring of the sufferer, the poorer the prognostic result.

Substantial research studies have recorded an association between the immunity infiltration in some mankind tumor types and prognoses and responses to treatments [28, 29]. Infiltration immunocytes are utilized as markers for the immune therapy reaction in multiple tumors [30]. Nevertheless, the role of each infiltration immunocyte type in tumor progression and the potential causal link remain elusive. Herein, our team established a prognosis model on the foundation of T-cell CD4 memory stimulated, monocytes, and neutrophils. The three immunocytes were related to OS of GC sufferers. In addition, survival assays confirmed that risk_high_ GC sufferers showed a shorter OS in contrast to risk_low_ sufferers, which was further confirmed by ROC assays. Finally, our team identified the association between the ceRNA prognosis model and the infiltration immunocyte prognosis model. Neutrophils were associated with PI15 in a positive way, which might reveal that the greater the expressions of such a gene, the greater the ICI level. Previously, PI15 was discovered to be dysregulated in several types of tumors, and its diagnostic and prognostic value was frequently reported [31–33]. However, the possible regulatory function between PI15 and immunity remained unknown, which needed further study. In addition, the potential of PI15 used as a biomarker for the depth of neutrophil infiltration needed to be further demonstrated in other experiments.

## 6. Conclusion

Overall, the two models can be used as reliable prognostic biomarkers for GC and can provide guidance for personalized therapy. More studies are needed for the demonstration of our findings using clinical experiments and in vitro and in vivo assays.

## Data Availability

The datasets used in this study are available from the corresponding author upon reasonable request.

## Abbreviations

GC: Gastric cancer
ICI: Immune cell infiltration
lncRNAs: Long noncoding RNAs
STAD: Stomach adenocarcinoma
Tregs: T cells
FC: Fold changes
ROC: Receiver operating characteristic curves
ceRNA: Competitive endogenous RNA
BP: Biological process
RS: Risk score
K-M: Kaplan–Meier
Tfh: T follicular helper.
